# Proteomic informed by transcriptomic for salivary glands components of the camel tick *Hyalomma dromedarii*

**DOI:** 10.1186/s12864-019-6042-1

**Published:** 2019-08-27

**Authors:** Chaima Bensaoud, Hajer Aounallah, Juliana Mozer Sciani, Fernanda Faria, Ana Marisa Chudzinski-Tavassi, Ali Bouattour, Youmna M’ghirbi

**Affiliations:** 1 Université de Tunis El Manar, Institut Pasteur de Tunis, LR11IPT03, Service d’entomologie médicale, 1002 Tunis, Tunisie; 2 Institute of Parasitology, Biology Centre, Czech Academy of Sciences, 37005 Ceske Budejovice (Budweis), Czechia; 3 Laboratório de Biologia Molecular, Instituto Butantan, Av. Vital Brazil, 1500, CEP, São Paulo, 05503-900 Brazil; 4 Laboratório Multidisciplinar de Pesquisa, Universidade São Francisco, Av. São Francisco de Assis, 218, CEP 12916-900, Bragança Paulista, São Paulo, Brazil

**Keywords:** *Hyalomma dromedarii*, Salivary glands, Proteome, LC–MS/MS, PIT

## Abstract

**Background:**

The hard tick *Hyalomma dromedarii* is one of the most injurious ectoparasites affecting camels and apparently best adapted to deserts. As long-term blood feeders, ticks are threatened by host defense system compounds that can cause them to be rejected and, ultimately, to die. However, their saliva contains a cocktail of bioactive molecules that enables them to succeed in taking their blood meal. A recent sialotranscriptomic study uncovered the complexity of the salivary composition of the tick *H. dromedarii* and provided a database for a proteomic analysis. We carried out a proteomic-informed by transcriptomic (PIT) to identify proteins in salivary glands of both genders of this tick species.

**Results:**

We reported the array of 1111 proteins identified in the salivary glands of *H. dromedarii* ticks. Only 24% of the proteins were shared by both genders, and concur with the previously described sialotranscriptome complexity. The comparative analysis of the salivary glands of both genders did not reveal any great differences in the number or class of proteins expressed their enzymatic composition or functional classification. Indeed, few proteins in the entire proteome matched those predicted from the transcriptome while others corresponded to other proteins of other tick species.

**Conclusion:**

This investigation represents the first proteomic study of *H. dromedarii* salivary glands. Our results shed light on the differences between the composition of *H. dromedarii* male and female salivary glands, thus enabling us to better understand the gender-specific strategy to feed successfully.

**Electronic supplementary material:**

The online version of this article (10.1186/s12864-019-6042-1) contains supplementary material, which is available to authorized users.

## Background

Hard ticks (Ixodidae) are unique among hematophageous arthropods, mainly for their long-term feeding that can last up to two weeks [1]. As they feed on different animals, the ticks come under pressure from their host’s immune system, which led to their fast evolution [2]. During the feeding period, the host reacts to the injury inflicted by the tick bite by starting a wide range of mechanisms to prevent blood loss [3]. The feeding ticks are thus exposed to host defense system components including, not only immune ones, but also platelet aggregation, coagulation, and inflammation components [3]. All of these responses are designed to disrupt tick feeding and cause its rejection from the host’s skin [4]. To avoid host defenses, ticks secrete saliva at the bite site that contain many biologically active molecules that display anticoagulation, antiplatelet, vasodilatory, anti-inflammatory, and immunomodulatory activities [3, 5, 6]. Ticks are thus able to feed on their hosts for days and even weeks without being disturbed by their immune system [6]. Not only do tick salivary compounds facilitate tick feeding, but they may also promote the survival and dissemination of infectious agents in the host [6]. The enhancement of pathogen transmission by tick saliva, called saliva-assisted transmission, or SAT, has been documented for several tick-pathogen associations [7]. However, only a few of the salivary proteins implicated in pathogen transmission have been identified and characterized to date [6]. Overall, deciphering the composition of tick salivary glands could lead to the discovery of new potential targets for developing vaccines for tick control and/or blocking pathogen transmission and new pharmacological compounds with anti-hemostatic, anti-inflammatory and antibacterial activities [8–10].

Recent advances in new technologies, mainly Next Generation Sequencing, or NGS, through transcriptomic and proteomic approaches, has led to insights into the molecular mechanisms involved in tick hematophagy, pathogen transmission, and tick-host-pathogen interactions [11]. In addition, these technologies have revealed the complexity of tick salivary composition, which has hundreds of different proteins including many that are novel [12]. Apart from being diverse, these molecules are multipotent and were shown to be endowed with pharmacological features [13]. Accordingly, several transcript and protein profiles of tick salivary glands were carried out in different stages of development, for both genders and feeding behavior [2, 14, 15]. These studies were also conducted to compare the salivary gland secretion of both hard and soft ticks [16].

More interestingly, sialotranscriptomic analyses improved proteomic studies of unknown genome species that seek to identify pharmaceutically active proteins [17, 18]. Previously, proteomic studies relied on the information in sequence databases and were thus able to detect only those proteins that were encoded by known genes. As such, the identification of proteins by tandem mass spectrometry posed a big challenge for a non-model species of which there is no available genome [19]. Proteomic informed by transcriptomic (PIT) approach helped to solve this issue by generating protein databases based on the expressed mRNA sequencing [17]. The possibility of using sample-specific databases derived from RNA-seq data revolutionized large-scale proteomics [17]. This approach was used in many studies related to tick saliva, especially with the expansion of tick sialotranscriptomic analysis using next-generation sequencing methods [11]. Although PIT was applied to the study of the saliva from several tick species, most saliva proteins and their impact on the host tick interaction remain unknown [18].

The camel tick, *Hyalomma dromedarii* Koch, 1844 (Acari: Ixodidae) is considered to be the most closely associated with camels and is well adapted to the deserts where tick hosts live [20]. It is a common species in regions with Mediterranean steppe vegetation and in desert climates in Africa, the Near East, Middle East, Far East, India, Mongolia, and Tibet [21, 22]. During its blood meal, this tick species is involved in transmitting an array of pathogens including *Theileria annulata* [23], *Rickettsia* [24, 25] and Crimean-Congo haemorrhagic fever virus [26, 27]. It can also transmit *Coxiella burnetii*, the agent of Q fever, to camels [28]. *Given this role in parasiting camels, H. dromedarii ticks were the object of* several studies seeking to characterize the molecules isolated from their salivary gland extracts and saliva. These studies focused on isolating those some molecules whereas the set of other molecules remain unknown. For this reason, we recently analyzed the sialotranscriptome of *H. dromedarii* using NGS technology, highlighting the wide range of transcripts expressed in the salivary glands of this tick species [29]. The functional annotation of transcripts has provided information on predicted protein families. However, questions remain about proteins that are actually present in the salivary glands of this tick species. We therefore carried out the first comprehensive proteomic analysis of *H. dromedarii* salivary glands. We used proteomics informed by transcriptomics to identify *H. dromedarii* salivary gland proteins in both genders using LC-MS/MS. This approach relies on the translation of the predicted genes from the sialotranscriptome of *H. dromedarii* tick and generates databases of predicted proteins that were used by MS/MS-spectra search engines to identify peptides contained in these salivary glands.

## Results and discussion

### *Hyalomma dromedarii* salivary glands proteome

As a hematophagous ectoparasite mainly of camels, *H. dromedarii* tick has developed a complex cocktail of bioactive molecules that target and neutralize the molecule secreted by the host camel that allows for successful parasitism [28]. Only few reports have explored *H. dromedarii* salivary glands. Compared to other hematophagous parasites, relatively little information exists about the molecular composition of *H. dromedarii* salivary glands [27, 28, 30].

**Fig. 1:**
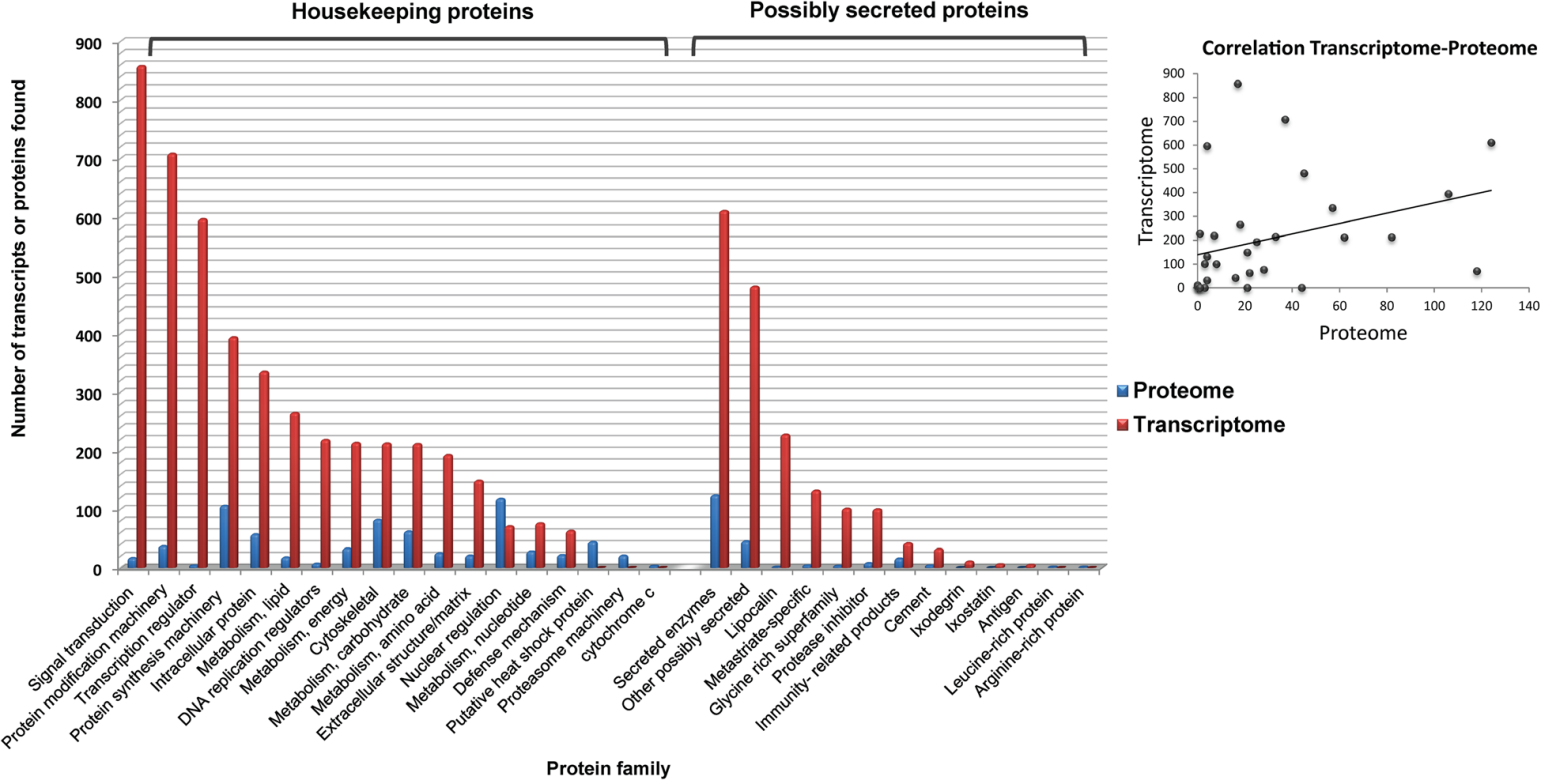
Correlation between protein families found in the transcriptome and proteome of salivary gland extracts from *Hyalomma dromedarii* tick

In our current work, we obtained salivary glands from partially engorged males and females of *H. dromedarii* collected from camels from south Tunisia. We can therefore consider our proteomic results to provide a qualitative description of *H. dromedarii* salivary glands components since the glands were stimulated during the tick’s attachment on the host. As the genome of *H. dromedarii* is not yet sequenced, we chose the PIT approach as the best way to identify the proteins of *H. dromedarii* salivary glands based on the recently published sialotranscriptome [29]. We identified 854 previously known proteins from the Acari database and an additional 257 proteins predicted from the transcriptomics data. A few studies have previously reported the use of transcriptomics to inform proteomics in other tick species including the *Dermacentor andersoni* [18]. The first proteomic studies addressing tick saliva and salivary glands date to the first decade of the twenty-first century [16, 31]. Each of these studies had a different focus: comparing the saliva of hard and soft ticks [16], partially and fully engorged *Rhipicephalus (Boophilus) microplus* [14], and sexual differences in the sialomes of *Rhipicephalus pulchellus* and *Ornithodoros moubata* [15, 32]. In addition to their different objectives, these proteomic analyzes also used different approaches to identify proteins following LC-MS/MS analysis [2, 14, 15, 32, 33]. We were able to identify a total of 1111 different proteins, far from the 15,342 proteins predicted from the sialotranscriptome [29]. Several protein families were identified in common in both proteome and transcriptome, while few were exclusively found in one of them (Fig. 1). Besides, our results showed a correlation (r) of 0.33, which appears to be a weak correlation between proteins found in the sialoproteome compared to those found in the sialotranscriptome.

Several hypotheses can explain the discrepancy between the transcriptome and proteome obtained in our study. Firstly, even though the transcriptome and the proteome have been analyzed on adult *H. dromedarii* at almost the same feeding stage, it is possible that the proteome corresponds to proteins translated from transcripts that existed before the transcriptomic analyzes [17]. In addition, although mRNA profiling is an important tool in gene expression analysis, transcript abundance does not always correlate with protein expression levels, mainly because of translation regulation where cells only translate proteins when they are needed [34]. That means that not all the sets of mRNAs in a cell are necessarily translated into proteins. Secondly, constraints limiting transcriptomics and proteomics may also lead to discrepancies. Indeed, transcriptomics data do not provide information regarding post-translational modifications, subcellular location, or protein degradation, which is not associated with a decrease of transcripts [35]. Furthermore, in transcriptomics, the number of reads may not directly represent the level of expressed proteins [15]. This is sometimes due to the mass assembly of reads that produces fragmented transcripts resulting in more than one coding sequence (CDS) for a single protein [35]. In addition, some peptides extended over such fragmented regions will not be identified. Other proteins exclusively expressed in ticks may not be identified because their sequences are not included in the database [11]. These proteins have been reported in several transcriptomic studies that revealed a large percentage of transcripts without any sequence similarity to any known protein sequence and therefore no known function [3]. Lastly, regarding proteomics, lower sensitivity in detection limits restricts the number of proteins that can be detected. Thus, less abundant proteins may not be identified.

**Fig. 2:**
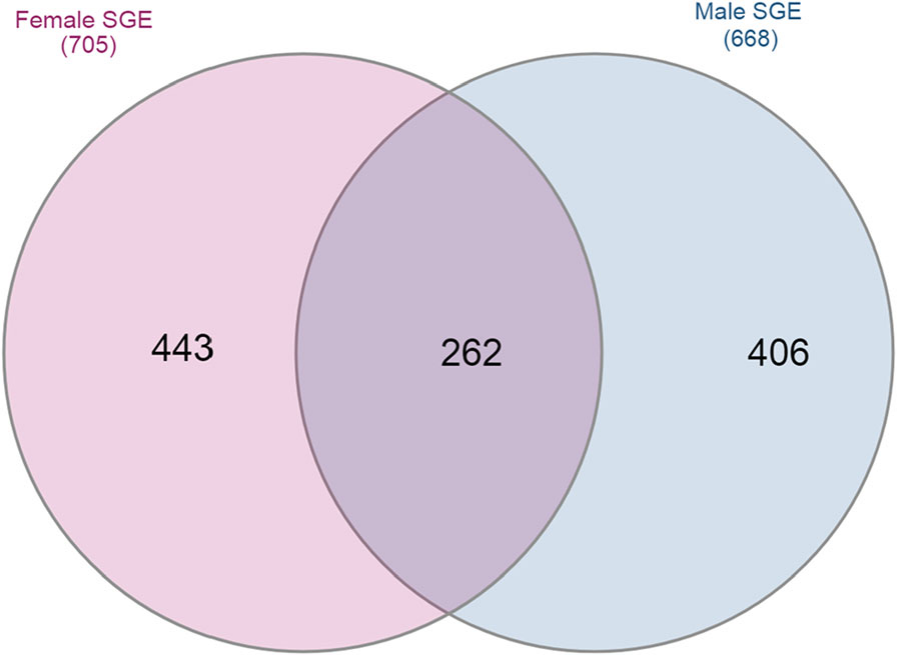
Venn diagram of SGE proteins identified in both genders of *Hyalomma dromedarii* ticks

According to our results, only 24% of the identified proteins (*n* = 262) were shared by both genders. In addition, among total proteins (*n* = 1111), 40% (*n* = 443) were found exclusively in females and 36% (*n* = 406) were found only in males (Fig. 2). These proteomic results concur with differences between the genders observed in the sialotranscriptome of *H. dromedarii* [29]. Indeed, transcriptomic data provided a global view of the gene expression profile in tick salivary glands while the proteomics analysis provides information regarding mRNAs that are actually translated into proteins.

We have identified almost the same amount of proteins in both genders but this does not rule out the difference observed in the transcriptomic study that is supported by the different protein identities shown in Additional file 1: Table S1 and Additional file 2: Table S2. Consequently, the so-called variation is assigned to the diversity of protein identities in the two genders. Such a difference was expected for several reasons (i) The anatomy and functions of Ixodidae salivary glands are known to be different between genders [36]. Females have three morphologically distinct acini types while males have additional specific acini [37]. Moreover, histological studies of tick salivary glands have described male-specific cells [38, 39] that were postulated to assist tick reproduction [40] (ii) Female ticks need days and even weeks to finish their extremely large blood meal on a single host where male ticks exhibit intermittent feeding during a shorter period of time and ingest small volumes of blood [41]. This means that the genders are exposed to different host defense constraints [41]. These differences between males and females was reflected in their proteomes, where the composition of their salivary glands is different [15, 42]. Several proteomic studies have therefore compared salivary gland compounds of both genders in hard and ticks [15, 32].

### Description and classification of proteins in salivary gland extract of *H. dromedarii*

**Fig. 3:**
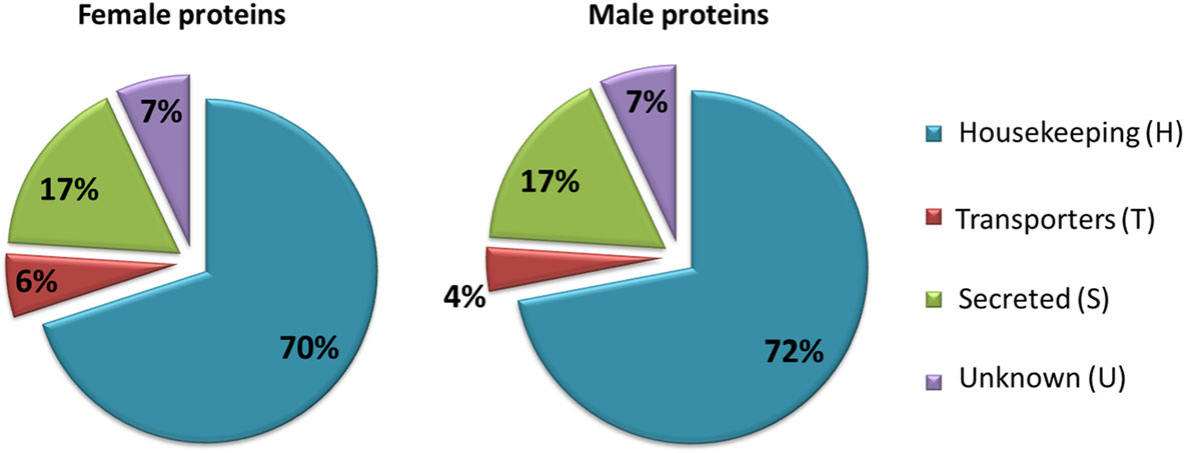
Distribution of proteins identified in *Hyalomma dromedarii* SGE of both genders according to their functions

According to protein function, the identified proteins were classified into four main groups as described in previous sialome studies [43]: housekeeping class (H), possibly secreted proteins (S), transporters (T), and unknown class (U) (Fig. 3). Of the set of identified proteins, 68% (*n* = 760) belonged to the H class, 19% (*n* = 207) to the S class, 8% (*n* = 89) to the U class and 5% (*n* = 55) to the T class.

### Housekeeping proteins (H class)

**Fig. 4:**
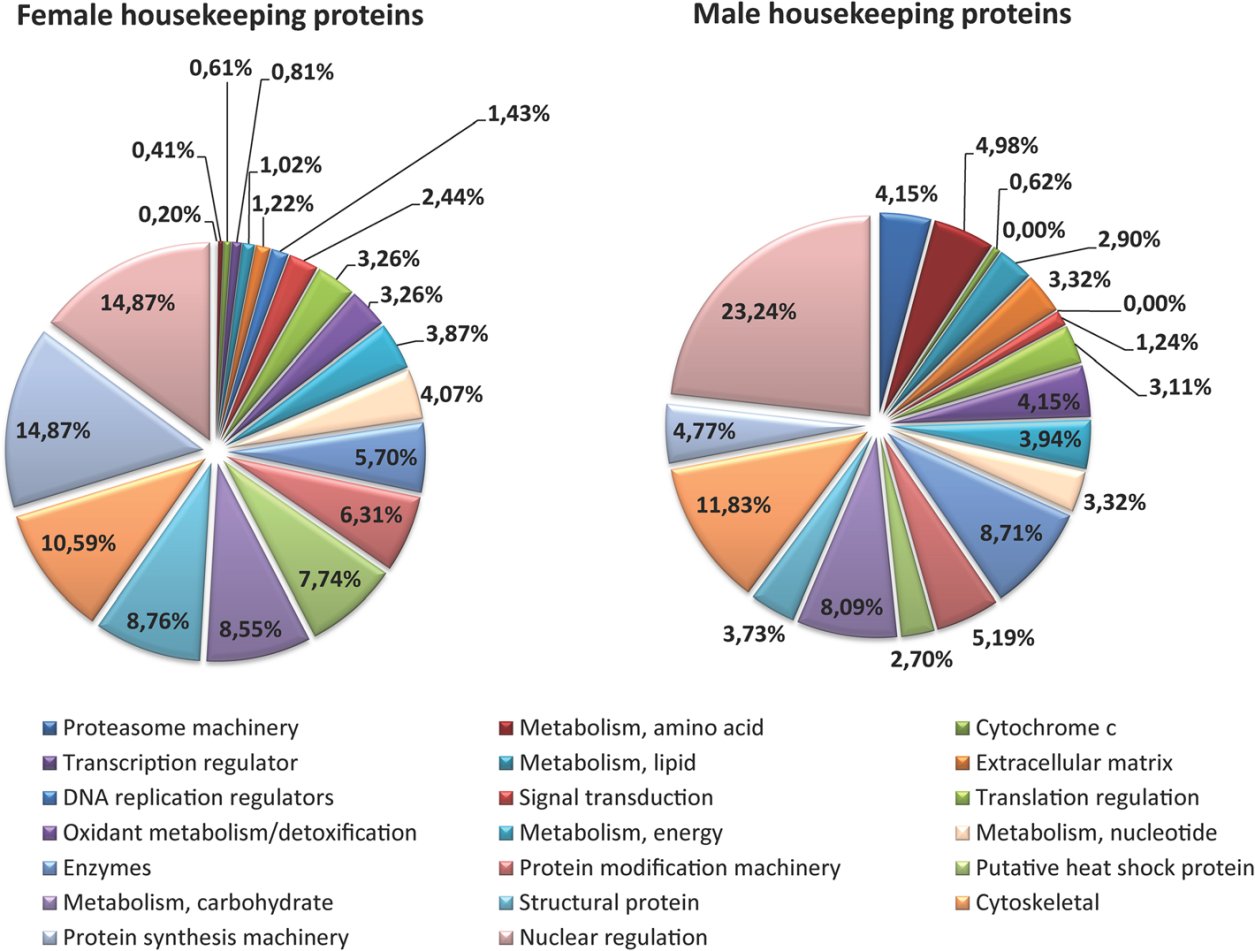
Classification of housekeeping proteins identified in *Hyalomma dromedarii* SGE of both genders, according to their functions and/or protein family

In *H. dromedarii*, 68% of the identified proteins for both genders were related mostly to the housekeeping class, which was the most abundant for both females (72%) and males (70%). This strongly corroborates the transcriptome data where transcripts coding for housekeeping proteins were the highly expressed ones [29]. As we are analyzing salivary gland extracts, we expected to find high amounts of housekeeping proteins, since they come from the leakage of intracellular components from degenerated or broken salivary gland cells [1]. Housekeeping proteins were defined as those required for the basal maintenance of tissues although it is also recognized that they might be secreted in the extracellular microenvironment especially since some of them were described in previous proteomic studies of saliva [32]. In this study, we organized housekeeping proteins into 20 groups by their functions and roles in the cell. All groups were the same in both genders except for transcription regulators and DNA replication regulators, which were only found in females. Proteins associated with nuclear regulation were most numerous in both genders. 15% in females and 23.2% in males of all proteins associated within the H class. These results suggest highly regulated gene transcription in the salivary glands of *H. dromedarii*. The second most abundant group for female ticks was the protein synthesis machinery group (14.9%), an expected result given the secretory nature of the organ. By contrast, cytoskeletal proteins (11.9%) represent the second most numerous group in males. Cytoskeletal proteins such as actin and tubulin were also found in females in large amounts (10.6%). These latter two proteins are largely conserved and were identified in almost all proteomes of other tick species [2, 32]. The remaining groups (*n* = 15), have nearly similar percentages as described in Fig. 4, and are involved, mainly, in the intracellular functions of tick salivary glands. As previously stated, sialomes have often noticed that some housekeeping intracellular proteins can be secreted by tick salivary glands where they play further extracellular function [44, 45]. Therefore, housekeeping proteins in *H. dromedarii* salivary glands may have further biological importance, particularly in the tick-host interface [14]. This could be explained by the presence of proteins in females (3.2%) and in males (4.2%) involved in the oxidant metabolism, as well as several enzymes linked to detoxification. For example, we identified superoxide dismutase, an essential enzyme involved in the mechanism of eliminating free radicals [46] and Glutathione S–transferase known for its catalysis of the conjugation of glutathione with several xenobiotic and endogenous substances [47]. These enzymes might be related to the decrease in the oxidative ability of phagocytes at the bite site, as reported in the saliva of the cattle tick *Rhipicephalus (Boophilus) microplus* [48]. Additional studies are thus needed to characterize their functions in the extracellular environment.

### Possibly secreted proteins (S class)

**Fig. 5:**
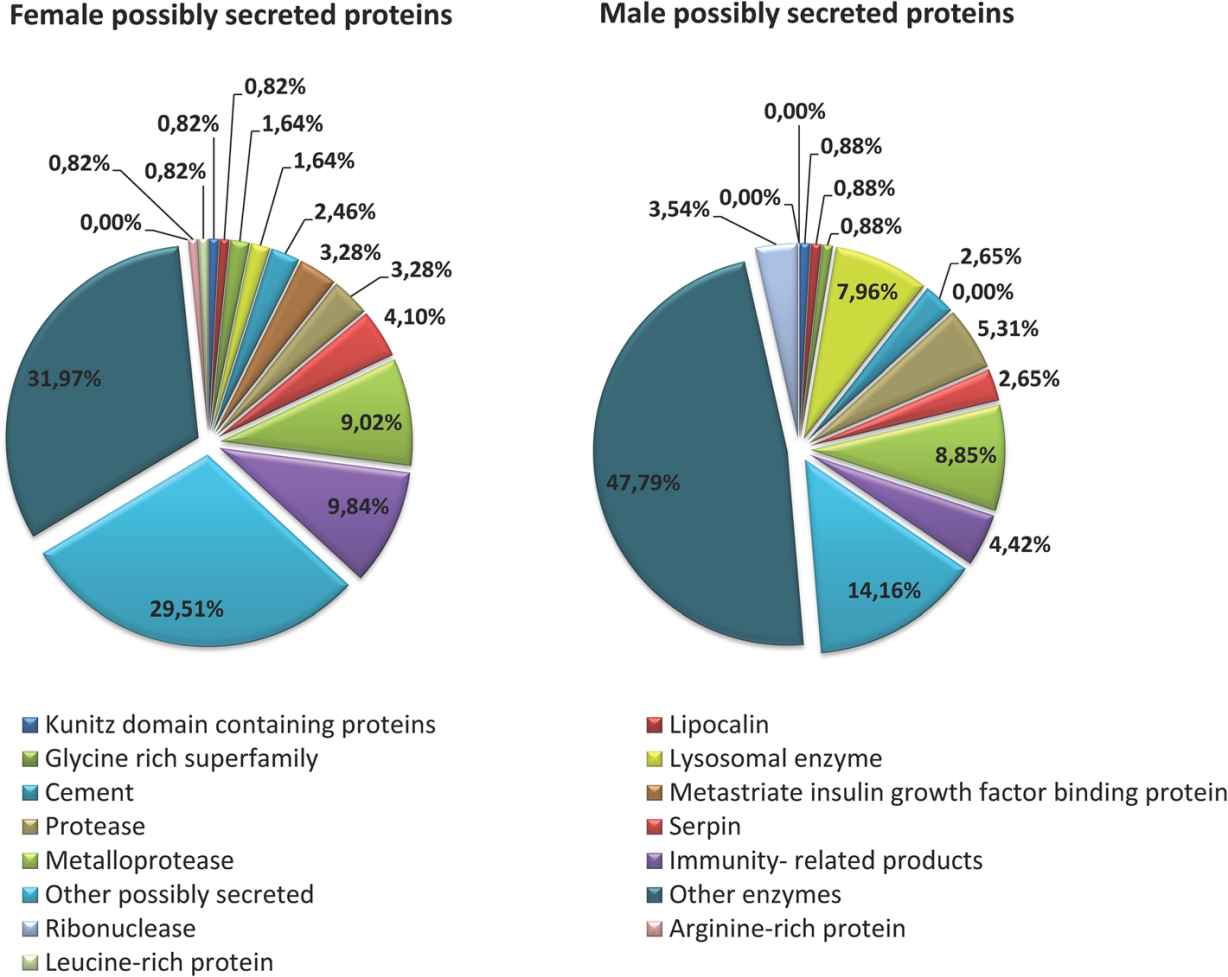
Classification of possibly secreted proteins identified in *Hyalomma dromedarii* SGE of both genders, according to their functions and/or protein family

Proteins of the S class were the second most numerous, with 19% from the set of identified proteins, and expressed equally (17%) in both genders. Secreted proteins were classified into 7 families as per a previous review [3]. Accordingly, most of the families described in Fig. 5 were identified in both genders, although a few were gender-exclusive. Our results showed a low number of the highly secretory protein families (2 Kunitz-type, 6 Serpins, and 2 Lipocalins) by contrast with the results observed in other hard ticks [2, 14, 15]. Moreover, no proteins from basic tail superfamily, mucins, Ixodegrin among others, were identified in the *H. dromedarii* proteome whereas these proteins are generally overexpressed in the proteomes of other ticks, including species from *Hyalomma* genus [2, 49]. It is important to emphasize that we cannot exclude the presence of these proteins in the *H. dromedarii* proteome given that we have previously identified these families in the transcriptome. It is probable that the heterogeneity observed for many of the principal secretory families in tick sialome [31, 43, 50, 51] is responsible for missing these proteins. Moreover, since we do not have the full repository of proteins from *H. dromedarii*, the lack of unidentified proteins can be explained by the absence of specific sequences in the databases needed for comparison.

As reported in Fig. 5, total enzymes represent more than 73% in males and 46% in females of S class proteins. Several enzymatic families have been identified in the proteome of both genders, including proteases, lysosomal enzymes and several other enzymes. In contrast, ribonucleases were identified exclusively in males. Metalloproteases were also among the identified enzymes with 9% from the set of S class proteins in both of the genders. These enzymes require a metal ion, usually Zn^2+^, to catalyze the hydrolysis of a peptide bond [52]. These proteolytic enzymes have been found throughout almost the entire evolutionary scale from bacteria to mammals [53, 54]. In snakes, they are a crucial component of venom and many of them cause their prey to hemorrhage [55]. In tick saliva, they were found to be associated with several physiological processes such as inflammation, fibrinolysis, blood protein digestion, vitellogenesis, immunomodulation, and pathogen transmission [52]. Given their abundance in tick saliva, several studies have focused on characterizing the metalloproteases of various tick species [56–58]. Other studies have even used these enzymes as antigens for anti-tick vaccines [52]. Other unusually secreted enzymes such as aconitate hydratase, and enolases were also identified in both genders. The latter enzymes have also been identified in the saliva of *Ornithodoros moubata* and shown to act as a profibrinolytic plasminogen receptor, most likely helping the tick to maintain the fluidity of host blood during feeding [59]. In *H. dromedarii*, enolases might be specifically secreted into the saliva where they could act as anti-hemostatic, anti-inflammatory or immunomodulatory compounds [60].

Protease inhibitor proteins including serpins and Kunitz-type proteins were detected in the proteome of both genders. These two super families were abundantly expressed in the sialotranscriptome of *H. dromedarii* but they were the lowest in the S class in the proteome.

Members of the Kunitz-type family are particularly well characterized as inhibitors of a large number of serine endopeptidases [36]. Most Kunitz domain containing proteins are serine protease inhibitors, although some also block ion channels [37]. Only 2 proteins containing Kunitz domains were found in the current proteomic analysis. Interestingly, these inhibitors have been characterized from previous tick sialomes, as acting upon thrombin, factor Xa, factor XIIa, trypsin and elastase [38]. This raises the suggestion they contribute to *H. dromedarii* saliva anticoagulant activity [39, 40]. Thus, additional studies are therefore needed to unravel the pharmacological properties of these proteins.

We identified 3 serpins in males and 5 in females, 2 of which were common for both genders. Serpins are one of the most important ubiquitous serine proteases inhibitors that ticks rely on to control host hemostasis and immunity [61]. Thorough functional characterizations of several individual recombinant tick serpins such as Iris and IRS-2 have revealed their anticoagulant, anti-inflammatory and immunomodulatory properties [62, 63]. It is therefore possible that the identified serpins assist the feeding process in *H. dromedarii* ticks, especially in females that remain attached for long periods of time to become fully engorged. Previous studies have shown that serpins are involved not only in avoiding the host defense system but also in reproduction by contributing substantially to the seminal fluid content in some insect species [61]. It was therefore not surprising that one of the 6 serpins that we identified was only found in males. This serpin may play a role in tick reproduction as did serpins in the seminal fluid of *Drosophila* [64, 65]. In depth studies of their functions are needed.

Like in the other tick salivary proteomes, proteins from Lipocalin family were found in *H. dromedarii* salivary glands. These proteins belong to a large family of proteins with low sequence conservation but characteristic structural features including an internal binding site [66]. Small hydrophobic molecules bind to this site and are generally transported to the extracellular environment [67]. Although they are among the most abundant transcripts in the transcriptome of *H. dromedarii*, lipocalins represent only 0.8% of the S class in both genders in the proteome. By contrast with our results, Lipocalins were the most abundant salivary proteins in the saliva proteome of *O. moubata* [32] and *R. microplus* [14]. Interestingly, the high content of Lipocalins in these tick species was suggested to be related to antihemostatic and immunomodulatory functions during feeding [68–70]. The amount of the identified Lipocalins does not appear to reflect the actual amount intended to be secreted into the saliva of *H. dromedarii*, making it likely that the functions of the Lipocalins we identified are similar to those proven in previous studies [70, 71].

Tick-specific proteins including Glycine-rich proteins were also expressed in the salivary glands of both genders. Together with cement proteins, they account for 4% of the S class in females and 3.5% in males. As glycine-rich do not produce suitable tryptic peptides [15], we believe that they might not all be identified in the proteome. Indeed, these proteins are known to play a crucial role in ticks. As a long-term blood feeder, *H. dromedarii* secrete a cement-like substance to strengthen their attachment to the hosts [37]. These proteins have been used as anti-tick vaccines isolated from other tick species [72–74]. Glycine-rich proteins may play roles other than in the tick-host relationship, especially in embryo development as was proven recently in *Rhipicephalus microplus* [72].

Tick-specific protein families were identified in the current study. Metastriate insulin growth factor-binding protein, a member of this family, was exclusively identified in female salivary glands of H. dromedarii. This proteins family was found in our transcriptomic analysis also [55], and was identified in several previous reviews of tick sialomes [56]. This family has two sets [57]. A shorter form includes only the IB domain, while the longer form has two additional domains, a Kazal domain and the SMART immunoglobulin C-2 type domain [57]. Interestingly, a human homolog proteins containing these three domains, named MC25, has several effects in tissue growth and differentiation [58, 59]. It has also been shown to inhibit vascular endothelial growth factor and keratinocyte growth [60, 61]. Therefore, proteins belonging to this family, identified in this proteome, could serve as binders of growth factors affecting angiogenesis, tissue repair, and immunity [57]. Deeper studies are needed to confirm these hypotheses.

The final difference is the immunity related proteins that account for 4.4% of possibly secreted proteins in males and 9.8% in female ticks. This difference may be explained by the different feeding behaviors of the two genders as previously described: *H. dromedarii* males do not remain attached to camels as long as females and therefore do not encounter the same host immune constraints. Both genders are thus expected to secrete a different arsenal of salivary molecules involved in encountering host defense. Interestingly, 6 of alpha 2 -macroglobulin (α2M) were identified in females compared to only one in males. These ubiquitous proteins have been identified in invertebrates and vertebrates. In vertebrates, α2M proteins have been found to regulate host cell apoptosis [75], inhibit several serum peptidases like thrombin [76], factor Xa [77] and kallikreins [78], mediate Tcell proliferation [79] and induce the proliferation and activation of macrophages [80]. Nevertheless, some studies on tick α2M have reported that they can intervene in inflammation and immunomodulation [79]. For this reason, we classified α2M proteins among the immunity related proteins in this study. It remains unclear, however, whether these α2M act as immunomodulators or as anticoagulants or as both: this role needs to be elucidated.

## Conclusions

Using proteomics informed by transcriptomics, we have generated the most comprehensive set of proteins detected in *H. dromedarii* salivary glands to date. Several protein families previously found in *H. dromedarii* transcriptome were identified in proteome. The identification of such proteins was indicative of a broad and complex proteome and concurs with the complexity of the previously described Ixodid sialomes. Our results provide new information regarding *H. dromedarii* salivary gland composition that may serve to guide further studies seeking to characterize each single protein using molecular, biochemical and pharmacological approaches. This study may provide new information on the tick-host relationship and offer new perspectives for drug discovery.

## Methods

### Experimental design and PIT workflow

**Fig. 6:**
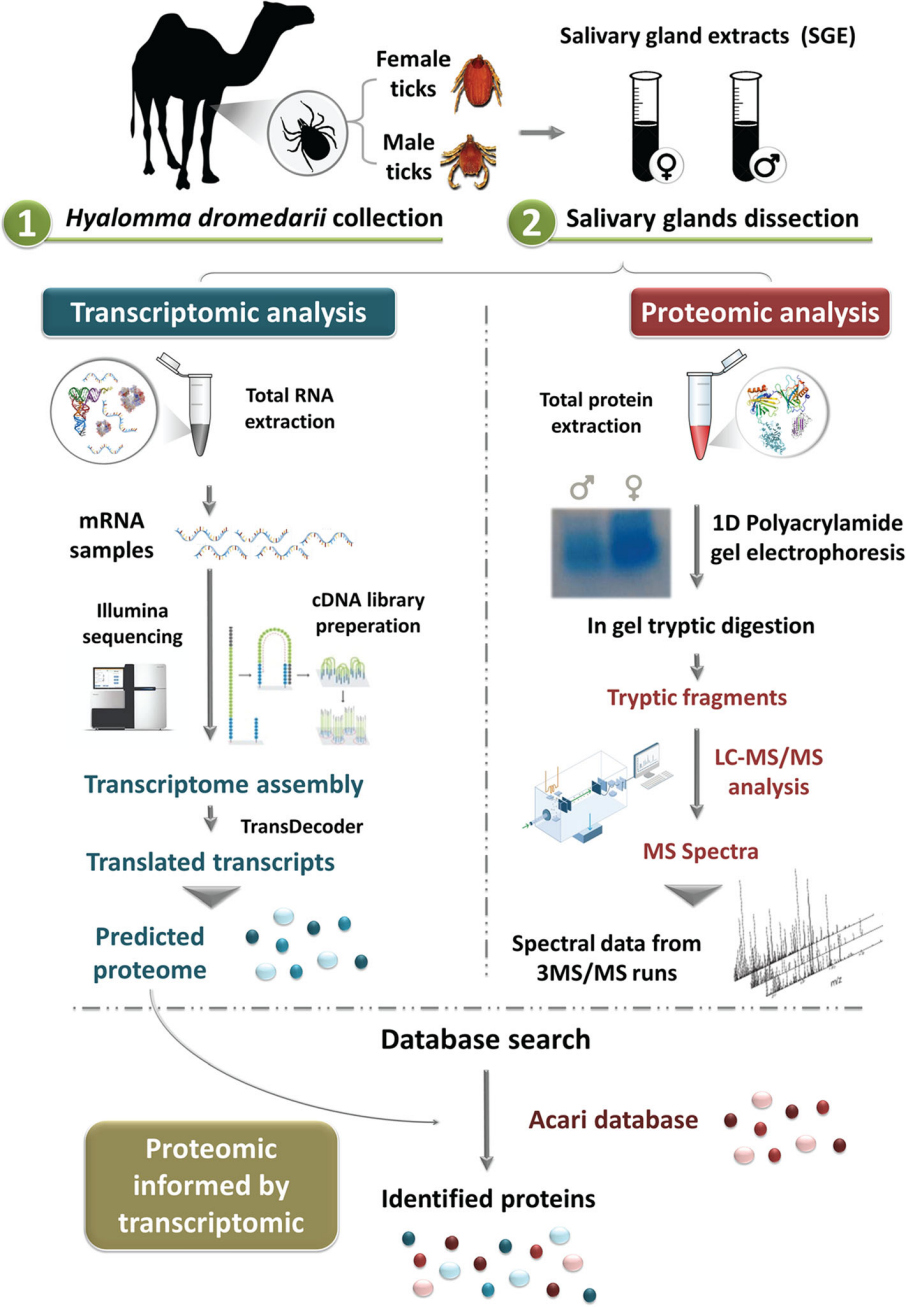
Experimental design; Proteomics Informed by Transcriptomics workflow

We used a PIT strategy to identify proteins from the salivary glands of both genders of *H. dromedarii* ticks, as summarized in Fig. 6.

### Ticks collection and salivary glands dissection

*H. dromedarii* ticks were collected from camels in the Saharan bioclimatic zone of southern Tunisia (33°25′908″ N, 009°00′952″ E). The camels were thoroughly inspected especially in their inguinal region and legs. Partially engorged ticks were removed manually and placed in flasks. Each tick was identified using a taxonomic key [81]. Within the first hour of collection, the ticks were washed and fixed in paraffin by their legs, after which lateral cuts were made using a scalpel to remove the scutum. The salivary glands of all the collected ticks were immediately teased away from other organs using ultra-fine forceps. Salivary gland samples were organized into pools according to the tick’s gender, one pool for female and one pool for male ticks. After dissection, the salivary glands were gently washed in ice cold phosphate buffered saline (PBS), pH 7.2 and stored at − 80 °C.

### Sample preparation and in gel digestion

The proteomic analysis was carried out in the SCSIE_university of Valencia Proteomics Unit, a member of ISCIII ProteoRed Proteomics Platform. Salivary glands were thawed, homogenized in PBS and centrifuged at 14000 rpm and 4 °C for 30 min. Salivary gland extracts (SGE) supernatants were pooled and the SGE protein concentration was determined according to the bicinchoninic acid method (BCA Protein assay, Pierce, Rockford, USA), as previously described [82]. Each solution (*~ 50* μg) was diluted by Laemmli SB (1x) to 25 μL. The samples (samples in 1x Laemmli buffer; 5 min a 95 °C) were loaded in 1D PAGE TGX AnyKD (Biorad, Gernany) at 20 mA for 1 h 30 min. The gel was stained with QC colloidal coomasie stain. The gel lanes were sliced and each slice was subjected to in-gel tryptic digestion. Samples were digested with sequencing grade trypsin (Promega, Gernany) as described elsewhere [83]. The digestion was stopped with TFA (1% final concentration). A double extraction with ACN was done. The peptide mixtures were concentrated by speed vacuum to 50 μL. Only 3 μg of the sample was analyzed for both females and males by LC-MS/MS.

### Liquid chromatography and tandem mass spectrometry (LC-MS/MS) analysis

Peptides resulting from tryptic digestion were loaded onto a trap column (NanoLC Column, 3 μ C18-CL, 350 μm × 0.5 mm; Eksigen) and desalted with 0.1% TFA at 3 μL/min during 5 min. The peptides were then loaded onto an analytical column (LC Column, 3 μ C18-CL, 75 μm × 12 cm, Nikkyo) equilibrated in 5% acetonitrile 0.1% FA (formic acid). Elution was carried out with a linear gradient from 5 to 35% B in A for 120 min. (A: 0.1% FA; B: ACN, 0.1% FA) at a flow rate of 300 nl/min. Peptides were analyzed in a mass spectrometer nanoESIqQTOF (5600 TripleTOF, ABSCIEX). The triple TOF was operated in information-dependent acquisition mode, in which a 0.25-s TOF MS scan from 350 to 1250 m/z was performed followed by 0.05-s product ion scans from 100 to 1500 m/z on the 50 most intense 2–5 charged ions.

### Database search

#### Protein pilot v4.5. Search engine (ABSciex)

The data obtained for each sample were analyzed and combined for a database search. Protein Pilot default parameters were used to generate a peak list directly from 5600 TripleTofwiff files. The Paragon algorithm of Protein Pilot was used to search the NCBI protein database with the following parameters: trypsin specificity, Iodoacetamidecys-alkylation, and the search effort set to rapid. To avoid using the same spectral evidence in more than one protein, the identified proteins were grouped based on MS/MS spectra by the Protein Pilot Progroup algorithm. Proteins sharing MS/MS spectra were therefore grouped regardless of the peptide sequence assigned. The protein within each group that can explain more spectral data with confidence was shown as the group’s primary protein. Only the proteins of the group for which there was individual evidence (unique peptides with enough confidence) were also listed, usually toward the end of the protein list. Proteins showing a Protein Pilot unused score above 1.3 were identified with greater than 95% confidence and considered significant.

#### Search engine used for protein identification

The raw files generated by Protein Pilot were used for protein searches using Peaks Studio Software (Bioinformatics Solutions Inc., Canada). Database searches (including a post-translational modification (PTM) search and a sequence variant search) were performed on the Acari database, constructed with proteins retrieved from the “Acari” term. Additionally, tandem MS and MS/MS spectra were searched against a recently published protein fasta database derived from *H. dromedarii* sialotranscriptome [29]. Searches were done with tryptic specificity allowing one missed cleavage and a tolerance on the mass measurement of 0.1 Da in MS mode and 0.1 Da for MS/MS ions. Carbamidomethylation of Cys was used as a fixed modification and oxidation of Met and deamidation of Asn and Gln as variable modifications. Proteins showing a score higher than homology or significance threshold were identified with greater than 95% confidence.

## Additional files

Additional file 1: **Table S1.** Classification of proteins identified in the salivary glands of *Hyalomma dromedarii* females. (XLSX 98 kb)

Additional file 2: **Table S2.** Classification of proteins identified in the salivary glands of *Hyalomma dromedarii* males. *Description of additional files. Accession:* protein ID in the database. *%coverage:* percentage Coverage_ The percentage of all the amino acids in the protein sequence that were covered by identified peptides detected in the sample. *-10logP:* the *P*-value is converted to -10log10 (*P*-value). In PEAKS, this value is denoted by -10lgP as lg is the ISO reserved notation for log10. By this conversion, a more significant match will have a higher -10lgP value. Additionally, a P-value of 1% is equivalent to -10lgP of 20. *#Peptides:* number of peptides identified following LC-MS/MS analysis for a single protein. *#Unique:* Unique peptides_peptides with the same amino acid sequence but different charges or with different modifications are grouped together and counted only once. *Avg.Mass:* the average mass of a single protein obtained by summing the average atomic masses of the constituent elements. *Description:* description of the identified proteins in the database. *Class:* housekeeping class (H); possibly secreted proteins (S); transporters (T); and unknown class (U). (XLSX 94 kb)

## Data Availability

The datasets supporting the conclusions of this article are included within the article and its additional files.

## Abbreviations

ACN: AcetonitrileA
BCA: bicinchoninic acid
CDS: Coding domain sequence
FA: Formic acid
LC: Liquid chromatography
MS: Mass spectometry
NGS: Next Generation Sequencing
PAGE: Polyacrylamide gel electrophoresis
PIT: Proteomic informed by transcriptomic
PTM: Post-translational modification
RNA-seq: Ribonucleic acid-sequencing
SAT: saliva-assisted transmission or
SGE: Salivary gland extracts
TFA: Trifluoroacetic acid
α2M: alpha 2 –macroglobulin
